# A novel role of HuR in ‐Epigallocatechin‐3‐gallate (EGCG) induces tumour cells apoptosis

**DOI:** 10.1111/jcmm.14249

**Published:** 2019-02-22

**Authors:** Wenxuan Jian, Shuhuan Fang, Tongkai Chen, Jiansong Fang, Yousheng Mo, Dongli Li, Sha Xiong, Wei Liu, Lei Song, Jiangang Shen, Yong xia, Qi Wang, Honghai Hong

**Affiliations:** ^1^ DME Center Institute of Clinical Pharmacology Guangzhou University of Chinese Medicine Guangzhou Guangdong Province China; ^2^ Department of Clinical Laboratory The Third Affiliated Hospital of Guangzhou Medical University Guangzhou Guangdong Province China; ^3^ School of Chinese Medicine The University of Hong Kong Pokfulam, Hong Kong China; ^4^ Department of Pathology and Laboratory Medicine Indiana University School of Medicine Indianapolis Indiana

## 1 INTRODUCTION

Hepatocellular carcinoma (HCC) is one of the most common malignancies worldwide.^1^ Inducing tumour cells apoptosis by chemotherapy is a common treatment modality for inoperable tumour.^2^ Some studies have shown that amyloid protein precursor (APP), which plays an important role in neuronal cells owing to their linkage with Alzheimer's disease,^3^ aberrantly altered in many types of cancers. However, the regulation of APP in tumour cells and its role in tumour cells remains unknown.‐Epigallocatechin‐3‐gallate (EGCG) has been shown to possess a wide range of pharmacological properties, including anti‐inflammatory and neuroprotective effects, which have been linked to the antioxidant/pro‐oxidant properties of its polyphenol constituents.^4^ Some studies have shown that EGCG inhibited hepatocellular carcinoma growth and induced apoptosis.^5^ We previously also found that EGCG induced neuroblastoma apoptosis via inhibition of amyloid precursor protein.^6^ However, little is known about the mechanism of EGCG on APP regulation in tumour.

The RNA‐binding proteins (RBPs) interact with other proteins and RNAs to form different ribonucleoprotein (RNP) complexes, which participate in the post‐transcriptional regulation of RNAs and which orchestrate the fate of those RNAs.^7^ Human antigen R (HuR, also known as ELAVL1) is an important RNA‐binding protein. Some studies reported that HuR correlated with patient outcome in liver cancer and interacted with lncRNA‐AK058003 to regulate proliferation and metastasis of hepatocellular carcinoma.^7^ Furthermore, in neurons, studies showed that HuD interacted with the 3’ UTRs of APP mRNA (encoding amyloid precursor protein) and BACE1 mRNA (encoding β‐site APP‐cleaving enzyme 1) and increased the half‐lives of these mRNAs.^8^ However, the role of HuR in APP expression and EGCG‐regulated APP expression in tumour is not clear.

In this study, we first indicate that the role of EGCG in APP and ADAM10 regulation is mediated by HuR, result in tumour cells apoptosis via HuR‐Erk1/2‐APP/ADAM10 pathway, adding a new dimension to EGCG‐mediated regulation of APP and ADAM10 in cancer cell apoptosis.

## 2 MATERIALS AND METHODS

### 2.1 Cell cultured

HepG2 and PC12 are cultured in Dulbecco's modified essential medium (DMEM) supplemented with 10% FBS and 1% Penicillin‐Streptomycin according to manufactures’ instruction (detailed in the “Supporting Information”).

### 2.2 EGCG treatment

See the “Supporting Information”.

### 2.3 MTT assay

MTT assay was performed according to manufactures’ instruction (detailed in the “Supporting Information”).

### 2.4 Clone formation assay

Clone formation assay was performed according to manufactures’ instruction (detailed in the “Supporting Information”).

### 2.5 Flow cytometry

Flow cytometry was performed according to manufactures’ instruction (detailed in the “Supporting Information”).

### 2.6 Total mRNA extract and qRT‐PCR assay

Total mRNA was extracted by Ultrapure RNA kit (CWBIO, China), and the concentration was measure by nanodrop2000 and reverse transcription to cDNA by Takara 5XPrimer Script RT Master Mix (for Real Time). qRT‐PCR was performed according to manufactures’ instruction (detailed in the “Supporting Information”).

### 2.7 Plasmid building and transfection

Plasmid building and transfection were performed according to manufactures’ instruction (detailed in the “Supporting Information”).

### 2.8 Western blot

Western blot was performed according to manufactures’ instruction (detailed in the “Supporting Information”).

### 2.9 mRNA stability study

mRNA stability study was performed according to manufactures’ instruction (detailed in the “Supporting Information”).

### 2.10 Statistical analysis

All data were expressed as the mean ± SEM. Statistical analyses were performed using Excel. Group comparisons are using Student's *t* test, 2 tails and double sample isovariances hypothesis. *P *<* *0.05 was considered significant.

## 3 RESULT

### 3.1 EGCG induces tumour cells apoptosis and regulates Bax and Bcl‐2 expression

We first evaluate the effect of EGCG on proliferation of HepG2 and PC12. The result showed that EGCG significantly inhibited proliferation of HepG2 and PC12 (Figure S1A, B). In addition EGCG obviously reduced the number of colonies formed of HepG2 and PC12 cells (Figure S1C,D) and distinctly induced tumour cells apoptosis (Figure S1E, F). To further validate whether EGCG actives the related proteins in the signal pathway of apoptosis, we analysed apoptosis‐related proteins Bax and Bcl‐2. Treatment with EGCG resulted in a dose‐dependent decrease in Bcl‐2 protein, and up‐regulation of Bax, leading to the increase in Bax/Bcl‐2 ratio (Figure S1G). In addition, similar results were obtained in the PC‐12 cells (Figure S1H).

### 3.2 EGCG reduces the expression of APP and ADAM10 via regulation of HuR in tumour cells

**Figure 1:**
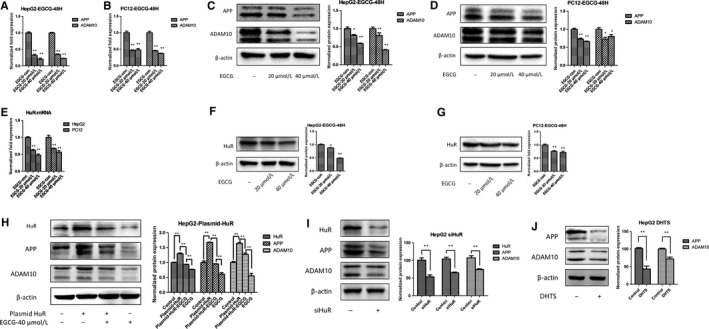
EGCG reduces the expression of APP and ADAM10 via regulation of HuR in tumour cells. A and B, The mRNA of APP and ADAM10 in HepG2 and PC‐12 treated with EGCG. Cells were treated with PBS, EGCG‐20 μmol/L or EGCG‐40 μmol/L for 48 h. **P <* *0.01. C and D, The level of APP and ADAM10 in HepG2 and PC12 was detected by Western blot. β‐actin acted as loading control. Cells were treated with PBS, EGCG‐20 μmol/L or EGCG‐40 μmol/L for 48 h. *P <* *0.05 and **P <* *0.01. E, The mRNA of HuR was reduced in HepG2 and PC12 treated with EGCG. **P <* *0.01. F and G, The protein level of HuR in HepG2 and PC12 treated with EGCG. β‐actin was used as loading control. Cells were treated with PBS, EGCG‐20 μmol/L and EGCG‐40 μmol/L for 48 h. H, The protein level of APP and ADAM10 in HepG2 and PC12 treated with EGCG or overexpression of HuR. β‐actin was used as loading control. *P <* *0.05, and **P <* *0.01. I, The HepG2 cells were transfected with siHuR for 48 h. The HuR, APP and ADAM10 level were detected by Western blot. **P <* *0.01. J, Detection of APP and ADAM10 level in HepG2 cells treated with the HuR inhibitor, Dihydrotanshinone I (DHTS, 5 μmol/L), for 24 h. **P <* *0.01

Next, we evaluated the expression of APP and ADAM10 in HepG2 and PC12 cells treated with EGCG. The results showed that the mRNA level of APP and ADAM10 were remarkably decreased compared with the control groups (Figure  1A,B). In accordance with the mRNA level, the APP and ADAM10 protein levels were also reduced (Figure 1C,D). We next evaluate the role of HuR in the regulation of APP by EGCG. The data showed that HuR level was significantly down‐regulated in HepG2 and PC‐12 cells (Figure 1E‐G). To evaluate the role of HuR in the regulation of APP and ADAM10 by EGCG, we overexpressed HuR in tumour cells. In Figure 1H, transiently transfected with HuR plasmids in tumour cells obviously eliminated the effect of EGCG‐down‐regulated expression of APP and ADAM10 (line 3 vs line 4). Besides, to exclude the artificial effect of overexpression, we also performed the experiment of knocking‐down HuR in the tumour cells. Silencing of HuR significantly decreased the APP and ADAM10 level (Figure 1I). Furthermore, we used a HuR inhibitor, Dihydrotanshinone I, also distinctly reduced the APP and ADAM10 level (Figure 1J). These results indicate that the reduction in APP and ADAM10 by EGCG was partly depended on HuR.

### 3.3 HuR regulates the APP processing by increasing the phosphorylation level of ERK rather than stabilizing the stability of APP mRNA

**Figure 2:**
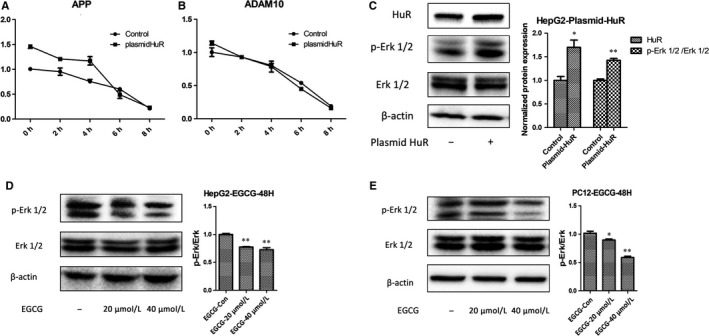
HuR regulates the APP processing by increasing the phosphorylation level of ERK rather than stabilizing the stability of APP mRNA. A and B, The relative mRNA expressions of APP and ADAM10 was detected by qRT‐PCR in HepG2 transiently transfected with HuR plasmids or treated with Actinomycin‐D. HepG2 was transiently transfected with HuR plasmids for 24 h, and treated with Actinomycin‐D for 0 h, 1 h, 2 h, 4 h, 6 h and 8 h. C, The level of p‐Erk1/2 was up‐regulated in HepG2 transiently transfected with HuR plasmids. β‐actin was used as reference. *P <* *0.05, and **P <* *0.01. D and E, The level of p‐Erk1/2 was decreased in HepG2 and PC‐12 treated with EGCG. β‐actin was used as loading control. *P <* *0.05, and **P <* *0.01

As HuR is an RNA binding protein via controlling the stability and translation of target mRNAs, we next estimate the role of HuR in mRNA stability of APP and ADAM10. Unexpectedly, the decrease rate of APP and ADAM10 has no difference between the experiment group and control group in tumour cell treatment with actinomycin D (Figure 2A,B). As Erk1/2 plays a key role in cell apoptosis and APP regulation, we hypothesize that HuR‐regulated APP and ADAM10 level through Erk1/2 signalling pathway. Indeed, overexpression of HuR enhanced the phosphorylation of Erk1/2 in tumour cells (Figure 2C). Furthermore, the phosphorylation of Erk1/2 was reduced in tumour cells treated with EGCG (Figure 2D,E).

## 4 DISCUSSION

(‐)‐Epigallocatechin‐3‐gallate (EGCG) is the most abundant polyphenolic extract in green tea, and has been shown to possess a wide range of pharmacological properties, including antitumour and neuroprotective effects.^9^ Some studies have shown that EGCG inhibited hepatocellular carcinoma growth and induced apoptosis,^5^ but the exactly molecular mechanism is not yet clear.

One of the key findings in this study is the critical role of amyloid protein precursor in EGCG‐induced HepG2 and PC‐12 cells apoptosis. Amyloid precursor protein (APP) is a member of the APP family of proteins, and different enzymatic processing leads to the production of several derivatives that are shown to have distinct biological functions.^10^ We previously found that EGCG induced neuroblastoma apoptosis via inhibition of amyloid precursor protein.^6^ In this study, we first demonstrated that EGCG induced HepG2 and PC12 apoptosis and regulated apoptosis‐related proteins Bax and Bcl‐2 expression. Further research showed that the effect of EGCG‐induced apoptosis was closely associated with APP, which was consistent with our previous study that EGCG induced neuroblastoma cells apoptosis via regulation of amyloid precursor protein.^6^ To our surprise, EGCG also decreased ADAM10 level, a member of the ADAM family involved in multiple cellular processes such as cell proliferation, differentiation, migration and invasion.^11^ Our results were in agreement with that silencing ADAM10 inhibits the growth of hepatocellular carcinoma cancer cells.^12^ These data indicated that EGCG induces tumour cells apoptosis via not only reduces APP, but also decreases ADAM10.

After establishing the critical role of APP in EGCG‐induced tumour cells apoptosis, we further identified HuR protein as the upstream mechanisms. Transiently transfected with HuR plasmids in tumour cells obviously eliminated the effect of EGCG‐down‐regulated expression of APP and ADAM10. These results indicated that the reduction in APP and ADAM10 by EGCG was partly depended on HuR. The key point for the next step was how HuR regulated APP and ADAM10. Unexpectedly, the decrease rate of APP and ADAM10 has no difference between the experiment group and control group in tumour cell treatment with actinomycin D which was used to inhibit de novo transcription.^13^ These data showed that the effect of HuR‐regulated APP and ADAM10 levels was not through stabilizing APP and ADAM10 mRNA stability. As Erk1/2 plays a key role in cell apoptosis and APP regulation,^14^, ^15^ we hypothesized that HuR regulated APP and ADAM10 level through Erk1/2 signalling pathway. Indeed, HuR enhanced the phosphorylation of Erk1/2 in tumour cells. Furthermore, the phosphorylation of Erk1/2 was reduced in tumour cells treated with EGCG, which was consistent with the reported that EGCG inhibits cognitive dysfunction through inhibition of ERK pathway.^16^ These results indicated that EGCG induced tumour cells apoptosis through the EGCG‐HuR‐ERK‐APP/ADAM10 pathway, rather than regulating HuR to stabilize the stability of APP and ADAM10 mRNA. In addition, since APP also acted upstream of Ras‐ERK signalling cascade,^17^ implying that ERK signalling regulated by APP probably in turn amplify the effect of EGCG to down‐regulating APP. This regulatory model was termed a ‘positive feed‐back loop’, which was universal in the process of cell growth controlled by growth factors such as PDGF/PDGFβ, EGF/EGFR, and VEGF/VEGFR.^18^, ^19^, ^20^

## ACKNOWLEDGEMENT

This study was supported by the National Nature Science Foundation of China (Grant No. 81403144, 81473740, 81673627); Guangzhou Science Technology and Innovation Commission Technology Research Projects, Open Tending Project for Construction of High‐Level University, Guangzhou University of Chinese Medicine (No. 34 and 118, 2017); the Characteristic Key Discipline Construction Fund of Chinese Internal Medicine of Guangzhou University of Chinese Medicine, the Guangzhou Science Technology and Innovation Commission Technology Research Projects (No. 201805010005), Project of Guangzhou Municipal Health Bureau (Grant No. 20171A011312), Guangzhou traditional Chinese medicine, integrated traditional Chinese and Western medicine science and technology project; Research project on elite talent training of Third Affiliated Hospital of Guangzhou Medical University; Foundation for Characteristic Innovation of Educational Commission of Guangdong (Grant No. 2016KTSCX011); Open Tending Project for Construction of High‐Level University, Guangzhou University of Chinese Medicine; Natural Science Foundation of Guangdong Province (2018A030310298) and Project of Educational Commission of Guangdong Province of China (2017KTSCX155).

## CONFLICTS OF INTEREST

The authors confirmed that there were no potential conflicts of interests.
