# Supplementary figures and images for: A novel role of HuR in ‐Epigallocatechin‐3‐gallate (EGCG) induces tumour cells apoptosis
Source: J Cell Mol Med. 2019 Feb 22;23(5):3767–71. doi: 10.1111/jcmm.14249 (PMC6484420; doi:10.1111/jcmm.14249)

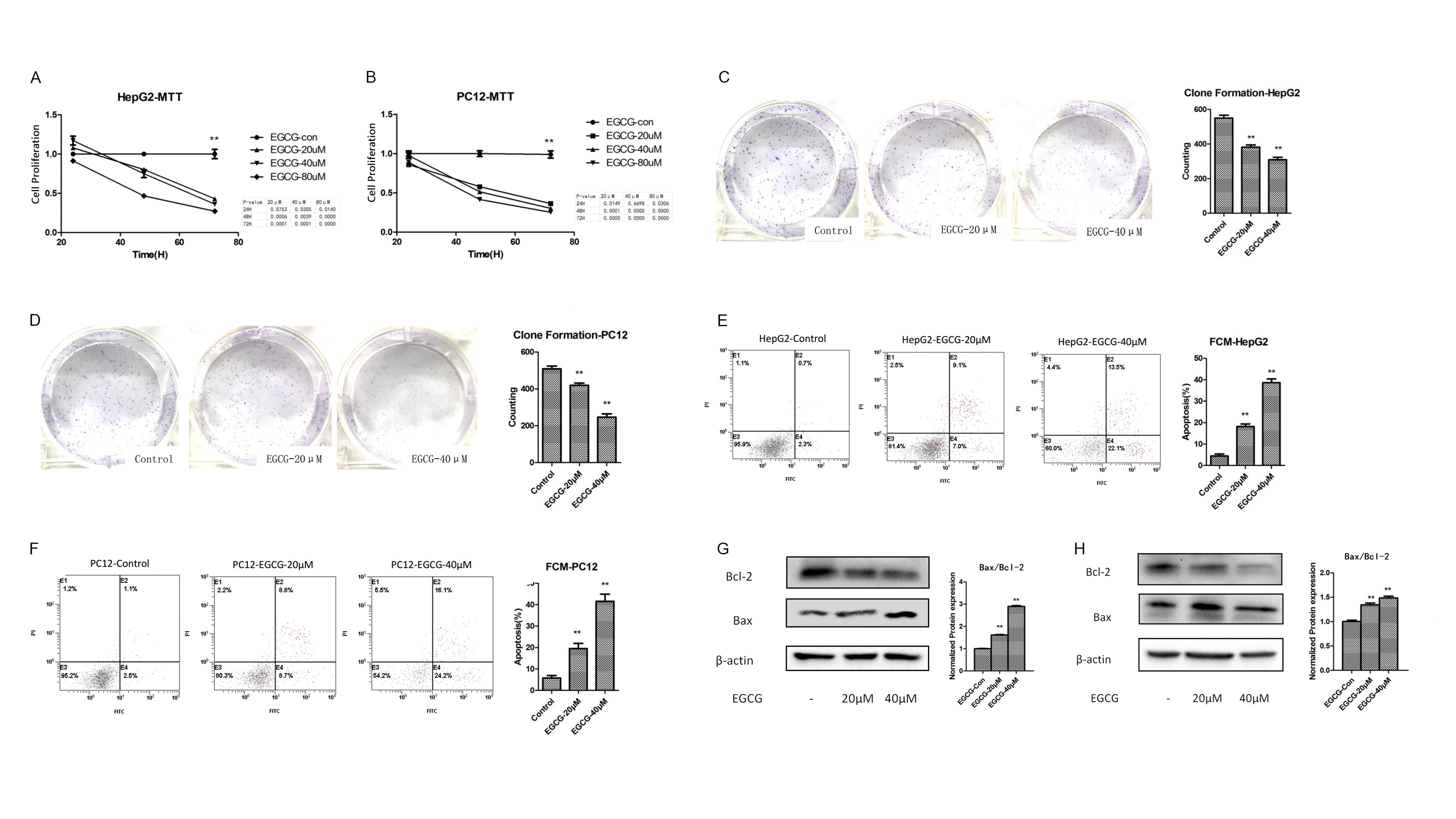

Supplement: Supplementary file 1 [file JCMM-23-3767-s001.tif]
