# Supplementary material for: A novel role of HuR in ‐Epigallocatechin‐3‐gallate (EGCG) induces tumour cells apoptosis
Source: J Cell Mol Med. 2019 Feb 22;23(5):3767–71. doi: 10.1111/jcmm.14249 (PMC6484420; doi:10.1111/jcmm.14249)
Supplement: Supplementary file 2 [file JCMM-23-3767-s002.docx]

**Supporting Information**

**Materials and method**

**Cell cultured**

HepG2 cells and PC12 cells are obtain from DME Center, Institute of Clinical Pharmacology, Guangzhou University of TCM. HepG2 and PC12 are cultured in Dulbecco’s modified essential medium (DMEM) supplemented with 10% FBS and 1% Penicillin-Streptomycin. The cells are incubated in 5%CO2 humidified air at 37℃ incubator. Media are replaced 2~3 times a week. HepG2 cells and PC12 cells are cultured in serum-free medium with DMEM for proper time for drug treatment, and then the cells were harvested and extracted for analysis.

**EGCG treatment**

EGCG is purchased from SIGMA(Cas:989-51-5).Once the cells are in a proper confluent, remove the medium, wash the cells with PBS gently, and replace the medium with DEME-EGCG(the concentration of EGCG is depends on the experiment),incubated in 5%CO2-37℃ incubator and prepares for the coming experiment.

**MTT assay**

Cells are cultured in the 96-well-plate in a density of 5,000 cells per well, and incubate with 100μl 10%FBS-DMEM for 24h.After 24h incubating, medium are removed and replaced with DEME with EGCG of different concentration for different time point. Medium are removed and replaced by 100μl 10%MTT-DMEM after the treatment and incubate for 4h.After the 4 hours MTT incubating, the medium are removed and replaced with 200μl DMSO and shake with 60rpm for 10 minutes in room temperature. And we measure the OD of every well with the microplate reader. We treated the control group as reference and use

$Normalized cell metabolic activity=\frac{\mathrm{eqODEGCG}-\mathrm{treatment}}{\mathrm{eqODcontrol}}$to measure the cell metabolic activity after EGCG treatment.

**Clone formation assay**

Cells are cultured in the 6-well-plate in a density of 6,000 cells per well, and incubate with 100μl 10%FBS-DMEM for 24h. After 24h incubating, medium are removed and replaced with DEME with EGCG of different concentration. Medium are replace every 2 days until the number of cell of each colony more than 50 and stop incubating. Medium are removed and cells are washed by PBS gently twice, and cells are immobilized by 4% Polyoxymethylene for 30 mins. Then cells are washed by PBS gently twice and staining with crystal violet for 30 mins. After staining, cells are washed by ddH_2_O gently and dry in dark at room temperature for 24 hours. Photos are captured under the white light and the cell colonies are count to indicate the clone formation ability.

**Flow cytometry**

Cell apoptosis is detected by Annexin V-FITC cell apoptosis kit (BEYOTIME.China), with FC 500 MPL(Beckman Coulter, US) . Cells’ medium are collected. Cells are washed by PBS and harvested with trypsin without EDTA in 1.5ml EP tube and washed in. Cells and the medium are collected in EP tube and centrifuge in 1000 rpm for 5min. Remove the medium and re-suspend cells with Annexin V-FITC buffer. Cells are incubate with V-FITC and PI in darkroom at the room temperature for 15-20mins, and detected with FC 500 MPL.

**Total mRNA extract and qRT-PCR assay**

Total mRNA is extracted by Ultrapure RNA kit(CWBIO，China),and the concentration is measure by nanodrop2000 and reverse transcription to cDNA by Takara 5XPrimer Script RT Master Mix(for Real Time). qRT-PCR is ran by Bio-rad CFX384 and TOYOBO 2X SYBR is used. The expression of target gene is measured by the Bio-rad CFX manager and GAPDH is used as reference. Primer sequences are described in table 1.

Normalized Fold Expression：$\triangle\triangle CQ=2^{(-\triangle CQ)}$，$\triangle CQ={CQ}_{target}-{CQ}_{GAPDH}$

Table1. Primer sequence

| Gene | Primer |
| --- | --- |
| HuR-homo | 5’-CCCTCTGGACAAACCTGTAGTC-3’  5’-GCCAACTTGTACATCAGCGG-3’ |
| APP-homo | 5’-GCTGGAGGTACCCACTGATG-3’  5’-TCTGCCACAGAACATGGCAA-3’ |
| ADAM10-homo | 5’-TGTACGCAGAGTATCTAACTGGA-3’  5’-GAAGCTTCCCACAAGGCAGT-3’ |
| GAPDH-homo | 5’-GCCCAATACGACCAAATCAGAG-3’  5’-GAAAGCCTGCCGGTGACTAA-3’ |
| HuR-CDS-homo | 5’-GGGGGTCTCTAGTG ATGTCTAATGGTTATGAAGAC-3’  5’-GCGGGTCTCGTGGG TTATTTGTGGACTTGTTGGT-3’ |
| HuR-Rat | 5’-CGCCCAAGCTCAGAGGTTAT-3’  5’-GACAAACCTGTGGTCTGATCC-3’ |
| APP-Rat | 5’-GCGGCAACAGGAACAACTTT-3’  5’-CTGCCGTCGTGGGAAACA-3’ |
| ADAM10-Rat | 5’-GGGCTGGGAGGTCAGTATGG-3’  5’-ACTGGTCCTCGTGTGAGACT-3’ |
| GAPDH-Rat | 5’-GGTTACCAGGGCTGCCTTCT-3’  5’-CTTCCCATTCTCAGCCTTGAC-3’ |

**Plasmid building and transfection**

The primer of HuR-CDS is designed by NBCI Blast. The cDNA of HuR-CDS is transfected to the DH5-α cell by Ruyilian Kit(Sidansai, China).The single colony is sent to the Invitrogen Biotechnology Co. Ltd to test the sequence. The sequence is compared to the HuR-CDS by NBCI Blast, which is to confirm the HuR-CDS is accurately transfected into the DH5-α cell. And the plasmid of HuR is extracted by the Endotoxin-free plasmid DNA purification(Macherey-Nagel). Plasmid of HuR is transfected to the HepG2 cell by FuGENE 6 Transfection Reagent(Promega).

**Western Blot**

The total cell protein is extracted with SDS lysate. The cytosolic protein is extracted with the Nuclear and Cytoplasmic Protein Extraction Kit (Beyotime, China). Concentration of protein is measured by BCA Protein Assay Kit (Beyotime, China). 10% or 12% Tris–glycine gels are used to electrophoresis depended on the molecular weight of protein. Protein are transferred to 0.22μm Immun-Blot PVDF(Bio-rad) in the method of wet electric transfection, washed in TBST, and blocked for 1h at room temperature in TBST containing 10% non-fat dry milk. The target antibodies are dissolved in 5% TBST, and the HRP antibody are dissolved in 3% TBST. Membranes were hybridized to the target protein antibody in 4℃ on the shaking table for overnight. Washed in TBST for 3 times 10 minutes and hybridize the proper HRP in room temperature for 1h and washed in TBST for 3 times 10 minutes. Densitometric analysis was done using the Bio-rad ChemDoc XRS+ with the software of Image Lab. The Normalized Protein Expression is counted as:

$Normalized Protein Expression=\frac{{\triangle Dens}_{Target}}{{\triangle\bar{\mathrm{Dens}}}_{Con}}$,$\triangle Dens=\frac{{Dens}_{x}}{{Dens}_{\beta-actin}}$

The antibodies are use as follows: HuR(abcam,ab200342), APP(CST,#2452), ADAM10(CST, #14194), Erk1/2(CST,#8544), p-Erk1/2(CST,#4370), Bax(CST,#14796), Bcl-2(CST,#3498), Caspase-3(CST,#9662)β-actin(CST,#3700), Anti-mouse IgG HRP-linked Antibody (CST,#7072), Anti-rabbit IgG HRP-linked Antibody (CST,#7071).

**mRNA stability study**

HepG2 cells are culture in 6-well plates, and transfect with the HuR-CDS or control vector. Actinomycin D (5 μg/mL final concentration) (Sigma-Aldrich, StLouis, MO, USA) was added at 24h after transfection. Total RNA was then harvested from cells at different time points (0, 1, 2, 4, 6 or 8 h) after actinomycin D treatment.

**Supplemental Figure legend**

**Figure S1. EGCG induces tumor cells’ apoptosis and regulates Bax and Bcl-2 expression**

(S1A and S1B) HepG2 and PC12 proliferation is tested by MTT assay. HepG2 and PC12 are treated with 20μM, 40μM and 80μM of EGCG for 24h, 48h and 72h. **P<0.001.

(S1C and S1D) Clone formation of HepG2 and PC12 treatment with EGCG. Clone formation and the amount of clones. **P<0.001.

(S1E and S1F) Apoptosis of HepG2 and PC12 treatment with EGCG are detected by FCM. Cells are treated with PBS, EGCG-20μM or EGCG-40μM for 48h, and evaluated the rate of apoptosis cells. **P < 0.01.

(S1G) The expression of Bax and Bcl-2 in HepG2 was detected by Western blot fellow with and Bax/Bcl-2 ratio analysis. Cells are treated with PBS, EGCG-20μM or EGCG-40μM for 48h. β-actin acted as loading control. **P < 0.01.

(S1H) The expression of Bax and Bcl-2 in PC12 was detected by Western blot fellow with and Bax/Bcl-2 ratio analysis. Cells are treated with PBS, EGCG-20μM or EGCG-40μM for 48h. β-actin acted as loading control. **P < 0.01.
